# Risk-Adjusted Impact of Administrative Costs on the Distribution of Terminal Wealth for Long-Term Investment

**DOI:** 10.1155/2014/521074

**Published:** 2014-08-12

**Authors:** Montserrat Guillén, Søren Fiig Jarner, Jens Perch Nielsen, Ana M. Pérez-Marín

**Affiliations:** ^1^Department of Econometrics, Riskcenter-IREA, University of Barcelona, Diagonal 690, 08034 Barcelona, Spain; ^2^ATP Pension Fund, Kongens Vaenge 8, 3400 Hilleroed, Denmark; ^3^Cass Business School, City University London, 106 Bunhill Row, London EC1Y 8TZ, UK

## Abstract

The impact of administrative costs on the distribution of terminal wealth is approximated using a simple formula applicable to many investment situations. We show that the reduction in median returns attributable to administrative fees is usually at least twice the amount of the administrative costs charged for most investment funds, when considering a risk-adjustment correction over a reasonably long-term time horizon. The example we present covers a number of standard cases and can be applied to passive investments, mutual funds, and hedge funds. Our results show investors the potential losses they face in performance due to administrative costs.

## 1. Introduction and Motivation

The administrative costs of saving and pension schemes have received increasing attention in recent years, at a time when a massive shift has been witnessed from actively to passively managed funds—often exchange-traded funds (ETFs)—that incur lower costs. The reason is obvious. Most studies of the performance of actively managed funds are unable to demonstrate their higher performance [1–3]. Mutual fund administrative fees are all expenses levied by a fund on its investors, covering investment management, administration, servicing, custody, and accounting services, among others [4]. OECD [5] recommends the promotion of low-cost retirement savings investments because of the huge impact of fee levels in terms of reductions in benefits for long contribution periods.

In this paper we show that the impact of administrative costs on terminal wealth can be substantial. We also propose a simple method for comparing median returns in the presence and absence of such costs, holding risk constant, and report that, for certain investment strategies, administrative fees can at times be twice as high as returns. Median values as opposed to expectations are used because the instrument we employ is the value-at-risk measure (i.e., quantiles).

While a good historical performance has been reported for a few actively managed funds, it is by no means straightforward to prove that this performance is anything but a random phenomenon (see, among others, [6, 7]). Let us assume for the time being that many savers want to avoid incurring additional administrative fees, especially if they are not certain of a higher performance.

When calculating the consequences of extra costs, a standard approach is used to employ a deterministic prediction of expected loss on returns, which is the difference between the expected returns on terminal wealth with and without the extra administrative costs. This is often sufficient to demonstrate the impact of administrative fees. A typical calculation, for example, might show that an additional one percent cost will mean that a quarter of a saver's pension disappears. This paper shows that while such calculations are highly thought as provoking, they may well be overly conservative, given that the saver may well incur costs greater than one percent. The difference between the expected returns on terminal wealth without administrative costs and with them is an estimate of only part of the impact, because extra costs influence the whole distribution of terminal wealth, not only first-order moments or location measures. This paper further examines this difference and provides a simple analytic expression for calculating the risk-adjusted impact of administrative costs on the median returns of terminal wealth. A deterministic prediction such as the difference between the expected or median returns of terminal wealth in the presence and absence of additional administrative costs fails to take the saver's risk aversion into account. It is tacitly assumed that the saver takes the same level of risk with or without these extra costs. Yet, we know that this is not the whole story. If a saver loses a quarter of his pension, then he will tend to be more risk averse with his remaining money; he will not want to lose much more. As such, a pension saver paying one percent in additional fees will tend to approach risky investments with more care than a saver who is not being charged these extra costs—this insight runs contrary to most descriptions in the literature. Another way of understanding this behaviour is in terms of a lost opportunity: the saver loses the opportunity to take risks with the one percent extra costs that he is apparently not prepared to lose. Such risky positions, however, give better median returns than less risky positions. As a result, the lost opportunity hits hard. We tacitly assume that greater risk implies better median returns and that greater risk implies that our risk measure reacts in the “right" direction. We use a simple quantile as our downside risk measure and we make the above assumptions explicit in our parameter space.

## 2. Background

Many authors have studied optimal consumption-investment problems [8–13]. Performance in terms of stochastic dominance has also been investigated. For instance, Huang et al. [2] find that funds that shift risk perform worse than those that maintain risk levels stable over time. They conclude that agency problems account for this behaviour; that is, fund managers are more interested in strategically shifting their risk levels to attract additional fund inflows than they are in concentrating on strategic investment. The authors use a holdings-based measure that they define as the difference between a fund's current holdings volatility and its past realized volatility. However, the standard deviation of the fund's actual return is a poor risk measure (see, for instance, [14]) that treats gains and losses symmetrically. Schuhmacher and Eling [15] analyze frequently used risk-based performance measures.

Many authors examine performance but do not focus on administrative costs [16–19]. Other costs paid by the investors, such as the price of an interest guarantee on with-profit policies, have been measured in terms of lost returns [20]. Cavenaile et al. [21] analyze the impact of illiquidity in hedge fund returns on their risk-adjusted performance and diversification potential. Eling [22] finds different levels of performance persistence in hedge funds, the results depending on the statistical methodology employed. The need to account for costs in long-term investments is, however, implicitly accepted [23, 24]. Costs associated to mortality-linked funds have been also investigated [25].

When studying mutual fund administrative fees, Khorana et al. [4] find that fees can vary substantially across funds and from country to country. They also show that larger funds tend to charge lower fees. Choi et al. [26] report that competition has not eliminated high-fee funds and that fees paid decrease with the financial literacy of the investor. Interestingly, subjects who choose high-fee funds feel they are making a mistake. Bergstresser et al. [27] quantify the benefits that investors purchasing mutual funds enjoy in exchange for the fees paid to brokers. All these studies serve to motivate the contribution reported here, that is, a proposal for a simple tool that can quantify the impact of administrative fees on performance while taking into account risk adjustment and long-term time horizons.

For lifetime investors, the importance of performance and transparency of pension schemes is widely recognized [28–31]. Small deviations in performance can become massive as a result of the long-term factor. Pension savers engage with their retirement savings for long periods of time, often over several decades. Current performance evaluation methods are usually too myopic and overlook persistence in performance, that is, the role played by time.

## 3. The Model

We assume a simple price process. Consider a Black-Scholes financial market, that is, a market with a constant risk-free rate of interest *r* and stock index (*S*_*t*_) following a geometric Brownian motion:

(1) $$\begin{array}{c} \frac{dS_{t}}{S_{t}}=\mu dt+\sigma dW_{t}, \end{array}$$

where *μ* is the expected return, *σ* is the volatility, and *W* is a standard Brownian motion.

Let *X* denote a wealth process and assume that wealth is invested at a constant proportion *α* in stocks and the rest at the risk-free rate. Assume further that* administration costs* are deducted continuously from the wealth process with a rate of *ν* proportional to the amount invested in risky assets. We therefore consider administrative costs (particularly, for custodial and accounting services) to be given by *αν*. The dynamics of *X* are thus given by

(2) dXtXt=αdStSt+(1−α)rdt−ανdt=rdt+α(μ−r−ν)dt+ασdWt.

The wealth process is a geometric Brownian motion with the well-known representation:

(3) $$\begin{array}{c} X_{t}=X_{0}\exp\left[\left(\alpha \left(\mu -r-\nu \right)+r-\frac{1}{2}\alpha^{2}\sigma^{2}\right)t+\alpha \sigma W_{t}\right]. \end{array}$$

Assume for ease of notation that *X*_0_ ≡ 1. More realistic conditions, including more general price processes with variance jumps or distributions with heavier tails, might be considered in the future. It could be argued that the stock price dynamics in our models are too simple to capture long-term effects. However, our aim is to study the model presented here first and, at a later date, to check the robustness of our results in more sophisticated settings. Additionally, wealth distribution may include the present value of pensions and future social security benefits together with some other forms of wealth. However, pension wealth is not perfectly fungible and some other household assets may also have liquidity constrains. Besides, differential tax treatment may also be applicable. Our approach does not consider this more general framework.

### 3.1. Quantiles

Since $W_{t}\overset{D}{=}\sqrt{t}U$ , where *U* is a standard normal variate, it follows that the *γ*-percentile, *q*_*γ*,*t*_, in the distribution of *X*_*t*_ is given by

(4) qγ,t=qγ(α,μ,σ,r,ν,t)=exp⁡[(α(μ−r−ν)+r−12α2σ2)t+ασtdγ],

where *d*_*γ*_ denotes the *γ*-percentile of a standard normal distribution.

Specifically, the median is given by

(5) $$\begin{array}{c} m\left(\alpha ,\mu ,\sigma ,r,\nu ,t\right)=\exp\left[\left(\alpha \left(\mu -r-\nu \right)+r-\frac{1}{2}\alpha^{2}\sigma^{2}\right)t\right]. \end{array}$$

We define the (geometric) rate of return

(6) $$\begin{array}{c} \rho \left(\alpha ,\mu ,\sigma ,r,\nu \right)=\alpha \left(\mu -r-\nu \right)+r-\frac{1}{2}\alpha^{2}\sigma^{2}, \end{array}$$

such that *m* = exp⁡(*ρt*). From (6), we can see that, given *α*, *r*, *μ*, and *σ* ^2^ fixed, the rate of return (*ρ*) is equal to a constant minus the administrative costs *αν*.

Note that, even in the absence of administrative costs, the rate of return is* negative* for sufficiently large values of *α*.

The expected value of *X*_*t*_, *E*[*X*_*t*_] = exp⁡[(*α*(*μ* − *r* − *ν*) + *r*)*t*], is always increasing in *α*. This reflects the fact that for high equity exposures the wealth distribution is highly skewed, the majority of the mass being close to zero and a long right tail.

Given the skewness of the wealth distribution, here we prefer to compare medians rather than means.

### 3.2. Parameter Space Restrictions

Our model parameters are *α*,  *μ*,  *σ*,  *r*,  *t* and the quantile level is *γ*. We restrict our parameter space to those parameters for which the median increases and the *γ*-percentile decreases when exposure to the risky asset increases.

The reason why we restrict our parameter space to this combination is to ensure that our assumptions are realistic. An increase in the median return is a necessary reward when downside risk increases in the distribution of *X*_*t*_. Thus, we assume that any risk-averse saver will only increase their exposure to the risky assets if the median return increases. We consider this restriction natural. Besides this property, we only consider nonnegative administrative costs (*ν*). All details are provided in Appendix A.

**Example 1 .**

For illustrative purposes, we present an example that we discuss in detail in the sections that follow. We use the capital market parameters *r* = 4%, *μ* = 8%, and *σ* = 20%. We also assume that *α* = 50% is invested in stocks and that *ν* = 0, so there are no administrative costs.

**Figure 1:**
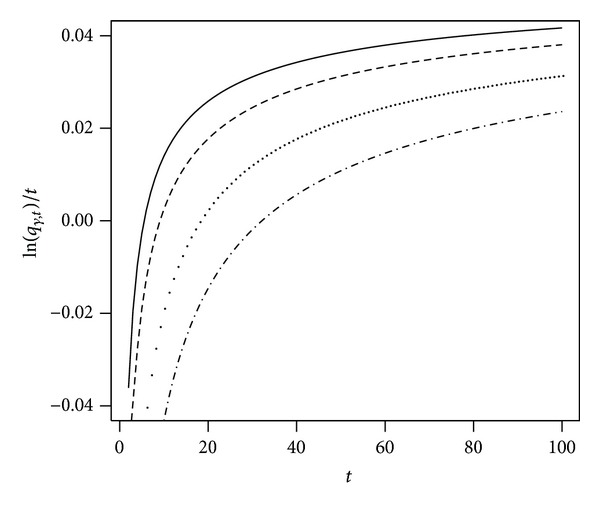
The plot shows ln⁡(*q*_*γ*,*t*_)/*t* as a function of *t* in our example. Parameters are *r* = 4%, *μ* = 8%, *σ* = 20%, *ν* = 0%, and *α* = 50%. We set *γ* = 10% (solid), 5% (dashed), 1% (dotted), and 0.1% (mixed).

Figure 1 illustrates the evolution of quantiles over time for some values of the probability level; that is, *γ* = 10%,  5%,  1% and 0.1%. We plot ln⁡(*q*_*γ*,*t*_)/*t* so we have a monotonic transformation of the quantile (log-scale of (4) divided by *t*).

Figure 1 shows that ln⁡(*q*_*γ*,*t*_)/*t* is negative in the initial period. Values of ln⁡(*q*_*γ*,*t*_)/*t* measure the loss in the lowest quantiles and capture the value-at-risk for the worst possible scenarios of the wealth process. After forty years, ln⁡(*q*_*γ*,*t*_)/*t* is clearly above zero, indicating that initial wealth has increased even for a situation that is at the 5% level of the downside tail.

In our example, all the conditions of the parameter space are satisfied for a time horizon of less than a hundred years.

## 4. Constant Risk of Terminal Wealth

We are interested in studying the effect of administrative costs on the median rate of return when fixing the level of risk in the wealth distribution. (A similar procedure could be applied to evaluate the effect of administrative costs on the expected rate of return.) The level of risk in the wealth distribution can be measured by a (low) percentile in the distribution, that is, a value-at-risk (VaR) measure.

Our procedure for analysing the impact of administrative costs is based on a constant risk factor. Thus, we examine the case for which we have no administrative fees, *ν* = 0, and calculate a risk measure for the distribution of wealth at time *t*. Then, we determine the equity exposure in the presence of administrative costs, *ν* > 0, that corresponds to the same risk as the given equity exposure with no administrative costs. Hence, we seek to find the reduction in equity exposure, Δ_*ν*_, such that the *γ*-percentile of the wealth distribution with equity exposure *α* − Δ_*ν*_ and administrative costs *ν*, *q*_*γ*_(*α* − Δ_*ν*_, *μ*, *σ*, *r*, *ν*, *t*), matches the *γ*-percentile of the wealth distribution with equity exposure *α* and no administrative costs *q*_*γ*_(*α*, *μ*, *σ*, *r*, 0, *t*).

Thus, we seek Δ_*ν*_, such that

(7) $$\begin{array}{c} q_{\gamma }\left(\alpha -\Delta_{\nu },\mu ,\sigma ,r,\nu ,t\right)=q_{\gamma }\left(\alpha ,\mu ,\sigma ,r,0,t\right). \end{array}$$

According to (4), we obtain the equivalent relation:

(8) ((α−Δν)(μ−r−ν)+r−12(α−Δν)2σ2)t  +(α−Δν)σtdγ =(α(μ−r)+r−12α2σ2)t+ασtdγ,

which after simple manipulations yields the quadratic equation:

(9) $$\begin{array}{c} A\Delta_{\nu }^{2}+B\Delta_{\nu }+C=0, \end{array}$$

where *A* = *σ* ^2^/2, $B=(\mu -r-\nu )-\alpha \sigma^{2}+\sigma d_{\gamma }/\sqrt{t}$ , and *C* = *να*.

It immediately follows from *(A.4)* that *B* ^2^ − 4*AC* ≥ 0. So, there is a solution for Δ_*ν*_, and it is given by

(10) $$\begin{array}{c} \Delta_{\nu }=-\frac{B-\sqrt{B^{2}-4AC}}{2A}. \end{array}$$

The other root, $-(B+\sqrt{B^{2}-4AC})/2A$ , corresponds to negative equity exposures.

**Example 1 (continued).**

**Figure 2:**
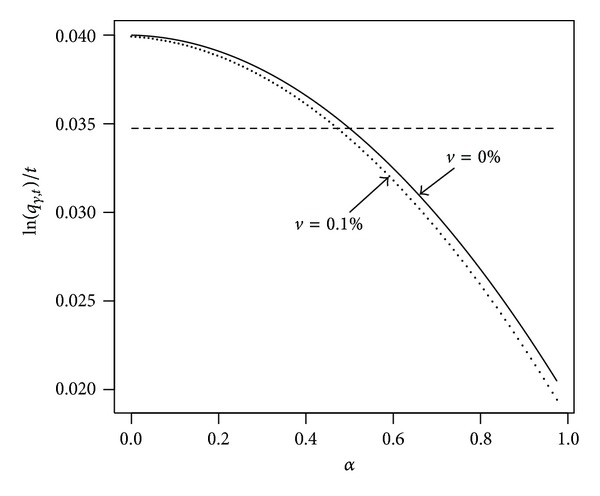
The plot shows ln⁡(*q*_*γ*,*t*_)/*t* as a function of *α* in our example, where *t* = 40. Parameters are *r* = 4%, *μ* = 8%, *σ* = 20%, and *γ* = 10%. We compare *ν* = 0% (solid) with *ν* = 0.1% (dotted). The horizontal dotted line indicates the value of ln⁡(*q*_0.1,40_)/40 when *α* = 50% and *ν* = 0%.

Figure 2 presents our example log-scale quantile divided by *t* as a function of *α* and the corresponding quantile when there are administrative costs. The graph shows that the level of investment in stocks *α* = 50% shifts to the left if we consider *γ* = 10% and *ν* = 0.1%. This means that when administrative costs exist, an investment equivalent in terms of our risk measure to one that has 50% invested in stocks and no administrative costs has a lower proportion invested in stocks, Δ_*ν*_ = 2.38%, for *t* = 40.

## 5. Rate of Return

Let *ρ*_*ν*_ be the rate of return with administrative costs *ν* and exposure *α* − Δ_*ν*_. It follows immediately from (6) that the difference between the rate of return *ρ* and *ρ*_*ν*_ is given by

(11) $$\begin{array}{c} \rho -\rho_{\nu }=\alpha \nu +\Delta_{\nu }\left(\mu -r-\nu \right)+\frac{1}{2}\Delta_{\nu }^{2}\sigma^{2}-\alpha \Delta_{\nu }\sigma^{2}. \end{array}$$

From the defining equation (8), it holds that

(12) $$\begin{array}{c} \alpha \nu +\Delta_{\nu }\left(\mu -r-\nu \right)+\frac{1}{2}\Delta_{\nu }^{2}\sigma^{2}-\alpha \Delta_{\nu }\sigma^{2}=\frac{-\Delta_{\nu }\sigma d_{\gamma }}{\sqrt{t}}, \end{array}$$

and we therefore arrive at the simple expression:

(13) $$\begin{array}{c} \rho -\rho_{\nu }=\frac{-\Delta_{\nu }\sigma d_{\gamma }}{\sqrt{t}}. \end{array}$$

In (13), as *d*_*γ*_ is negative, the reduction in the rate of return is positive. Given our assumptions, it is not surprising that the inclusion of administrative costs brings a reduction in the geometric rate of return (note that the geometric rate of return is based on the median of the wealth distribution) for *X*_*t*_. However, note that Δ_*ν*_ depends on *t*. As a consequence, the reduction in the rate of return attributable to administrative costs does not necessarily tend to zero as *t* increases. In certain specific cases, it might be constant, but in general it is increasing in *t*.

In order to understand expression (13) we need to find a simple first-order approximation. This should enable us to see the dependence of *ρ* − *ρ*_*ν*_ on *t* or its rate of convergence to a constant as a function of *t*.

### 5.1. First-Order Taylor Expansion of Lost Rate of Return

Our aim is to examine the behaviour of the reduction in the rate of return as a function of *ν*. We derive a second-order Taylor expansion of Δ_*ν*_ at *ν* = 0. Since by definition Δ_0_ = 0 we have

(14) $$\begin{array}{c} \Delta_{\nu }={\frac{\partial \Delta_{\nu }}{\partial \nu }|}_{\nu =0}\nu +\frac{1}{2}{\frac{\partial^{2}\Delta_{\nu }}{\partial \nu^{2}}|}_{\nu =0}\nu^{2}+o\left(\nu^{2}\right). \end{array}$$

Differentiating (12) with respect to *ν* yields

(15) (α−Δν)+∂Δν∂ν(μ−r)+Δν∂Δν∂νσ2 −∂Δν∂νασ2=−(∂Δν/∂ν)σdγt,

and by inserting *ν* = 0 and rearranging the terms, we obtain

(16) $$\begin{array}{c} {\frac{\partial \Delta_{\nu }}{\partial \nu }|}_{\nu =0}=\frac{\alpha }{-\left(\mu -r\right)+\alpha \sigma^{2}-\sigma d_{\gamma }/\sqrt{t}}. \end{array}$$

Similarly, by differentiating (12) twice and inserting *ν* = 0 we obtain

(17) ∂2Δν∂ν2|ν=0=σ2(∂Δν/∂ν|ν=0)2−(μ+r)+ασ2−σdγ/t=α(ασ2−2(−(μ−r)+ασ2−σdγ/t))(−(μ−r)+ασ2−σdγ/t)3.

Using (13) and after a few more simple manipulations, it follows that

(18) ρ−ρν=−Δνσdγt≈−(∂Δν∂ν|ν=0ν+12∂2Δν∂ν2|ν=0ν2)σdγt=ναβ+12∂2Δν∂ν2|ν=0σdγtν2,

where

(19) $$\begin{array}{c} \beta =\frac{1}{1+\left(\left(\left(\mu -r\right)/\sigma \right)-\alpha \sigma \right)\sqrt{t}/d_{\gamma }}. \end{array}$$

From these expressions, the first- and second-order effects of *ν* can be seen on the reduction in the rate of return.

Note that our chosen parameter space insures that *β* is positive and strictly larger than 1. The term $\left(\left(\left(\mu -r\right)/\sigma \right)-\alpha \sigma \right)\sqrt{t}/d_{\gamma }$ is negative and larger (see *(A.3)*, where *d*_*γ*_ is negative, because *γ* < 50%) than −1.

An interesting feature of our first-order approximation to the lost rate of return *ρ* − *ρ*_*ν*_ is

(20) $$\begin{array}{c} \rho -\rho_{\nu }\approx \alpha \nu \beta . \end{array}$$

So, we can concentrate on *β*, which is a multiplier, to obtain a simple, easy-to-use, first-order approximation of the impact of administrative costs (*αν*) on the lost rate of return. Moreover, we note that *β* tends to one as *t* decreases, but depending on the parameters and in the medium horizon of *t*, *β* can be quite large, as becomes evident in the illustrations presented in the next section. So, when measuring the impact of administrative costs in terms of the multiplier *β*, which is larger than 1, we see that it can grow rapidly. (The second-order effect is in the same direction.) According to (20) we also see that for analyzing the effect of administrative costs on the median rate of return it is necessary to fix the level of risk factors. For illustration, Example 1 uses different values of the probability level *γ*; namely, *γ* = 10%,  5%,  1%, and 0.1%. Additionally, the values of *r*, *μ*, *σ*, *ν*, and *α* also need to be fixed, according to realistic scenarios for generic investment products. In Section 6, parameter values for low, medium, and high volatility scenarios are proposed and used in the illustration of the impact of administrative costs.

### 5.2. First-Order Approximation of the Opportunity Cost

We turn now to concentrate on *β* and think of this multiplier as a way of assessing the impact of administrative costs on the rate of return lost in a feasible time horizon for investing. When *β* > 1 we can formally define the opportunity cost up to first-order as

(21) Cop=β−1=−(((μ−r)/σ)−ασ)t/dγ1+(((μ−r)/σ)−ασ)t/dγ=−(μ−r−ασ2)σdγ/t+(μ−r−ασ2)  .

We see that *C*_*op*_ depends on the risk premium (*μ* − *r*) and is increasing with the percentile *γ*, because when *γ* increases then *d*_*γ*_ increases. Note again that since we concentrate on downside risk, we take *γ* < 50%, and then *d*_*γ*_ is negative. The opportunity cost also depends on the particular combination of stock exposure *α* and volatility *σ* ^2^. Additionally, we also see the interrelations between the opportunity costs *C*_*op*_ and the investment period. Thus, the most interesting result is that the opportunity cost *C*_*op*_ also increases with the investment period *t* and can be extremely large when *t* increases. Note that, according to Condition 2 in Appendix A (where the parameter space is defined), the investment period cannot be infinitely long. The maximum value for *t* equals [*σd*_*γ*_/(*μ*−*r*−*ασ* ^2^)]^2^ and then the opportunity cost tends to infinity. Recall that *d*_*γ*_ is negative.

It is our belief that the size of the opportunity cost has perhaps been underestimated in the literature to date on the grounds that it may dilute over time. We have shown that the opportunity cost depends on the period of investment. This particular result is of critical importance to pension savers, who make long-term decisions. We show in the illustrations that follow in reasonably long-term horizons of forty years the opportunity cost effect can be quite substantial. Our result provides a possible explanation as to why too often most funds present a poor performance, especially for long-term investment horizons.

**Example 1 (continued).**

In our example, we consider an investment horizon of *t* = 40 and we use as a risk measure the 10%-percentile; that is, *γ* = 10% and thereby *d*_*γ*_ = −1.28. With *α* = 50% invested in stocks and no administrative costs the rate of return is *ρ* = 5.5%. Assuming administrative costs of *ν* = 0.1%, recall that Δ_*ν*_ = 2.38% and then we find that *ρ* − *ρ*_*ν*_ = 0.1%, and so the reduction in the rate of return is 0.1%.

Using the Taylor expansion we have

(22) $$\begin{array}{c} \rho -\rho_{\nu }\approx \nu \alpha \beta , \end{array}$$

where *β* = 1.9744. Thus, the first-order rate of return is reduced by an amount that is equivalent to about twice the administrative costs (*αν* = 0.05%). We examined the precision of the first-order approximation in our example, the details of which are provided in Appendix B.

**Figure 3:**
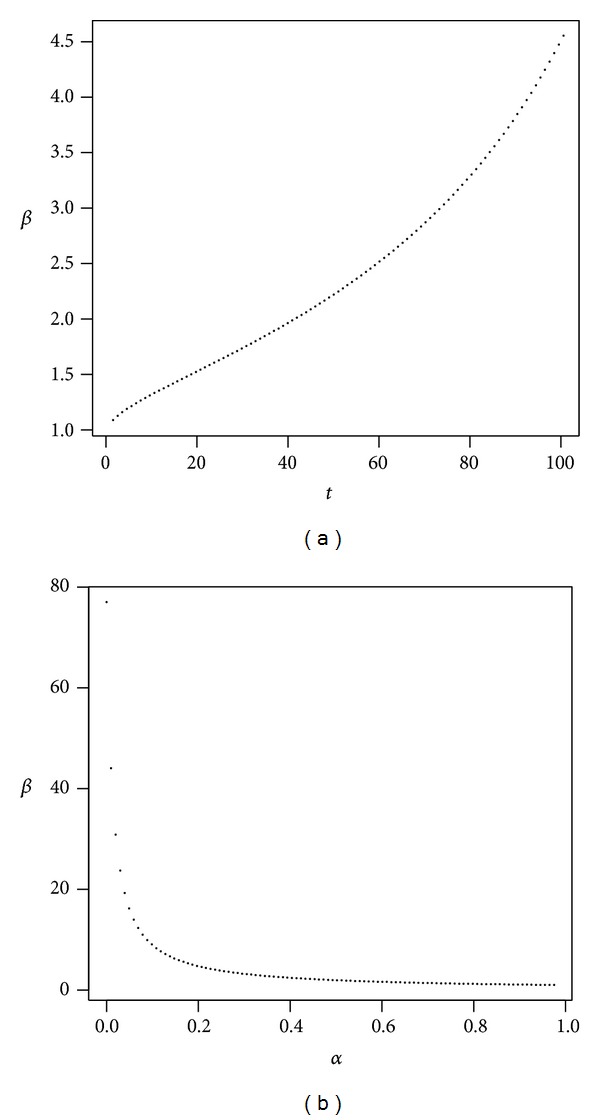
The plot shows *β* as a function of *t* (a) and *α* (b) in our example. Parameters are *r* = 4%, *μ* = 8%, *σ* = 20%, *ν* = 0.1%, *γ* = 10%, and *α* = 50% (a) and *t* = 40 (b).

Below we consider the two main reasons why administrative fees constitute a burden to the investor. First, we analyze the impact of administrative costs on the reduction in the rate of return, *β*, as a function of time. Second, we present the dependence of the opportunity costs on the proportion invested in stocks.  Figure 3(a), which presents *β* as a function of time, shows that while the impact increases, it is not very great in the first few years of the investment; however, the reduction in the rate of return increases substantially in the long term.  Figure 3(b) shows that as the percentage invested in stocks rises, the relative reduction or the cost of opportunity becomes smaller.

## 6. Illustrations

In this section we consider three possible standard scenarios for generic investment products. We assume different scenarios governing the mean and variance in market returns. (In practice, similar scenarios can be found in passive investment, active investment mutual funds, and hedge funds.) The model and methodology used for the approximations of the impact of administrative costs are the same for each scenario.

Parameter values for the selected scenarios are presented in Table 1. We begin our illustrations with a standard scenario characterized by low volatility (*σ* = 0.16) in the returns. If volatility is only moderate, the mean level of stock investment return is usually also quite low. We assume very low administrative fees in this case. Next, we examine a situation in which expected returns are higher, as are administrative costs. We distinguish three possible medium volatility scenarios (*σ* = 0.25) in this case, as described in Table 1. Finally, we examine the impact of administrative costs on investments under an assumption of high volatility (*σ* = 0.75). Here volatility is much higher than in the previous scenarios and expected returns are also higher, even though the proportion invested in stocks in this example is considerably smaller than that in the previous two cases. Administrative costs in this high volatility scenario are also higher than the fees charged in the low and medium volatility scenarios.

Our aim is to assess the impact of administrative costs in terms of the reduction in the rate of return and to do this we measure the multiplier *β* in (20). The last column in Table 1 presents the *β* values for each set of parameter cases. It can be seen that *β* > 1 in all cases, often around 2 and sometimes even much higher. The reduction in the rate of return due to the presence of administrative costs (*αν*) is roughly twice (*β* ≈ 2) the amount of these costs in the *t* = 40 year horizon for the low volatility and the medium volatility scenarios. For optimistic medium volatility investments in the high volatility scenario considered here, the reduction in the rate of return is much higher than twice the amount of administrative costs.

In the next section we discuss the evolution in *β* as a function of time and in *β* as a function of *α*. To simplify the presentation, we select a maximum time horizon of one hundred years. The size of *β* shows the enormous burden of administrative costs due to the cost of opportunity.

### 6.1. Low Volatility

**Figure 4:**
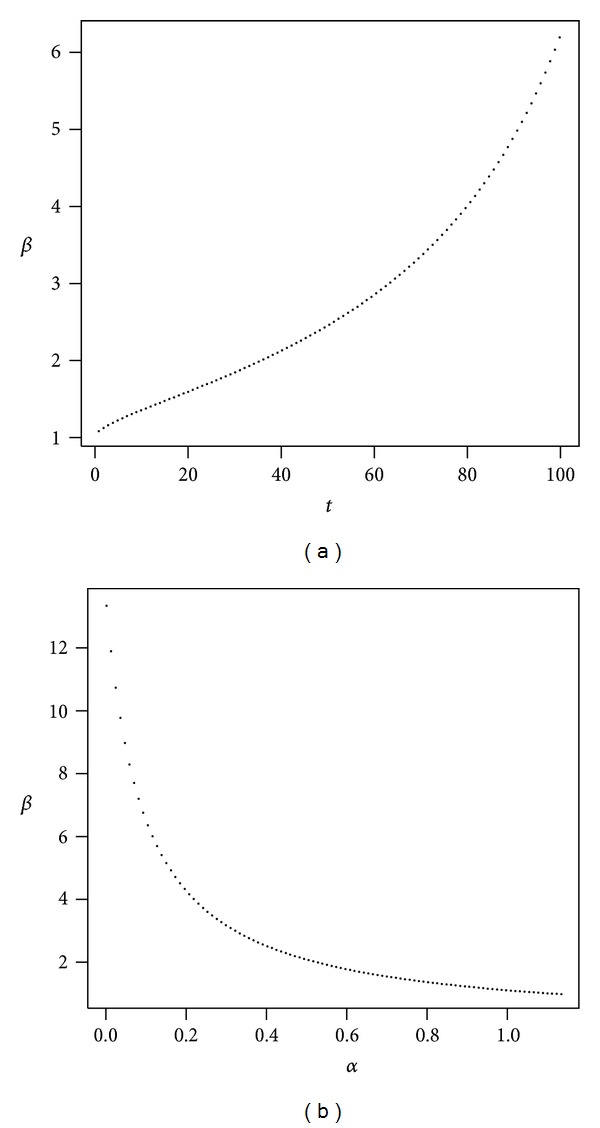
Low volatility of returns. The plot shows *β* as a function of *t* with *α* = 50% (a) and *β* as a function of *α* with *t* = 40 (b). Parameters are *r* = 4%, *μ* = 7%, *σ* = 16%, *ν* = 0.1%, and *γ* = 10%.

In the first scenario, we consider low volatility as described in Table 1.  Figure 4 presents the evolution of *β* as a function of time and *β* as a function of *α*.

For this case we observe that the opportunity cost due to the presence of administrative costs is well above 1 when the proportion invested in stocks is low.  Figure 4(b) shows that if the proportion invested in stocks is less than 20%, then *β* is above 4, so the opportunity cost increases more than threefold in a time horizon of 40 years.  Figure 4(a) shows that long-term investment horizons only exacerbate the situation, since *β* keeps growing with time. It might be argued that, in our choice of parameters for a standard, low volatility investment of low expected returns, administrative costs are also low and so the opportunity cost is not relevant. However, this conclusion is drawn on relative terms and the costs can be much more significant in absolute terms or in real wealth.

### 6.2. Medium Volatility

**Figure 5:**
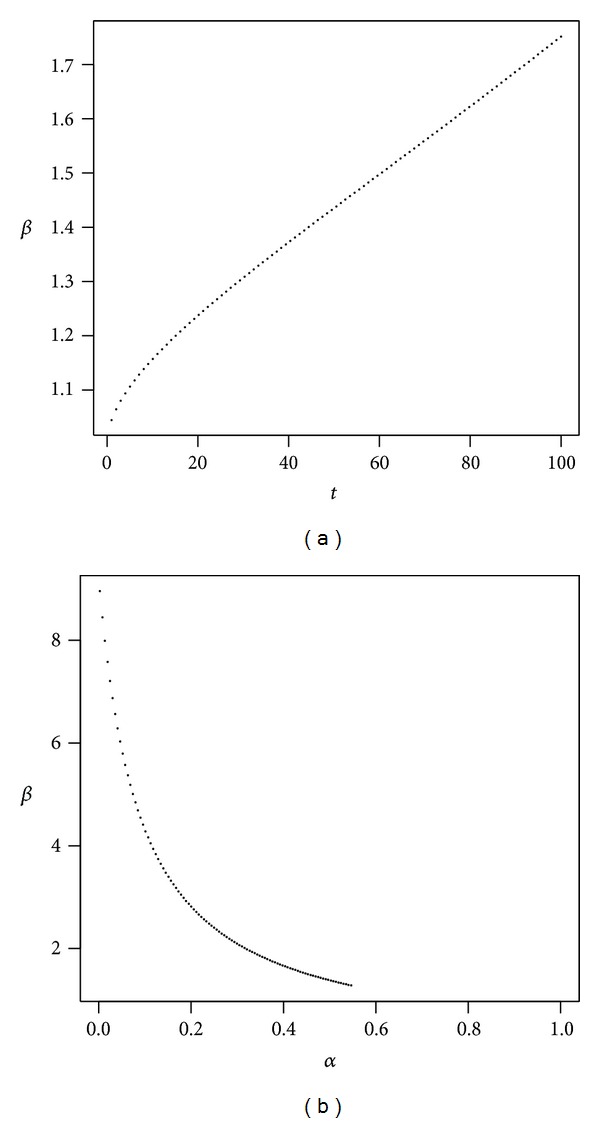
Medium volatility of returns (pessimistic). The plot shows *β* as a function of *t* with *α* = 50% (a) and *β* as a function of *α* with *t* = 40 (b). Parameters are *r* = 4%, *μ* = 8.5%, *σ* = 25%, *ν* = 1.1%, and *γ* = 10%.

**Figure 6:**
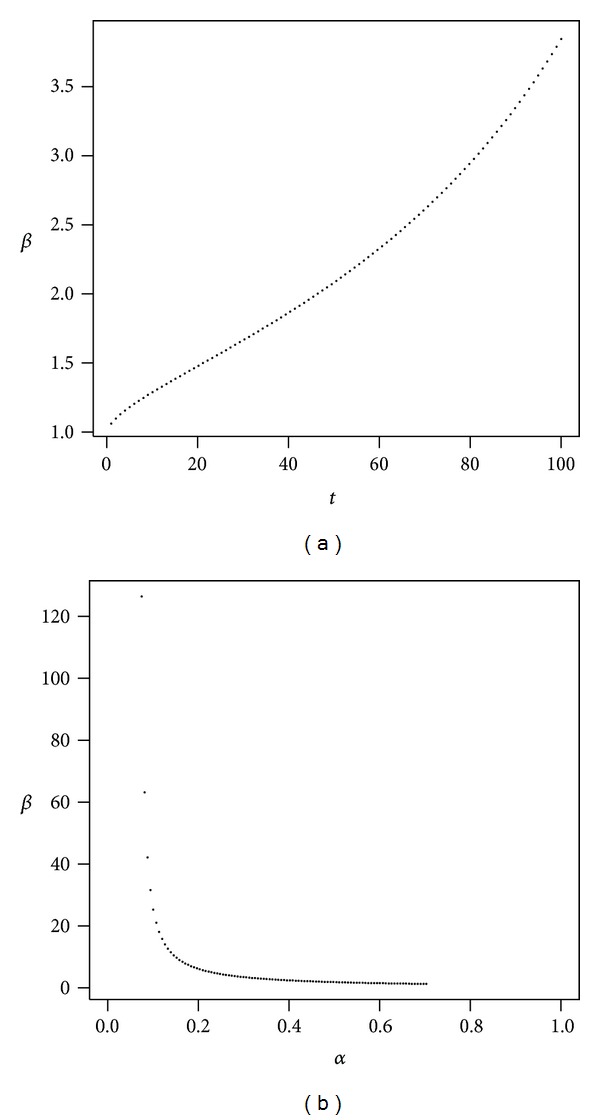
Medium volatility of returns (breakeven). The plot shows *β* as a function of *t* with *α* = 50% (a) and *β* as a function of *α* with *t* = 40 (b). Parameters are *r* = 4%, *μ* = 9.5%, *σ* = 25%, *ν* = 1.1%, and *γ* = 10%.

**Figure 7:**
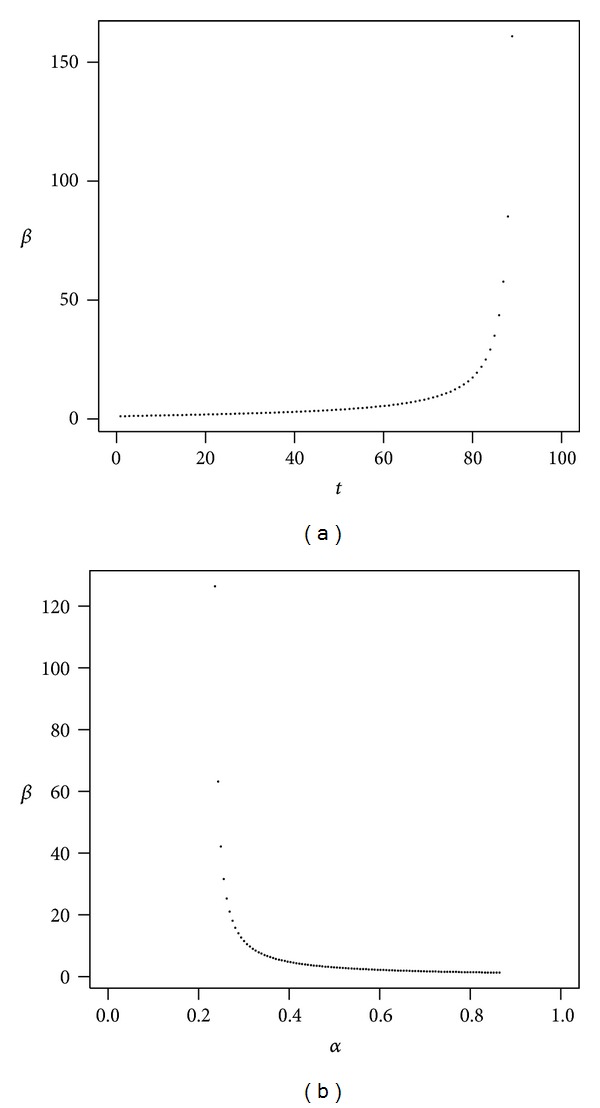
Medium volatility of returns (optimistic). The plot shows *β* as a function of *t* with *α* = 50% (a) and *β* as a function of *α* with *t* = 40 (b). Parameters are *r* = 4%, *μ* = 10.5%, *σ* = 25%, *ν* = 1.1%, and *γ* = 10%.

We also considered a scenario characterized by medium volatility and medium administrative fees. Figures 5, 6, and 7 present the evolution of *β* as a function of time and *β* as a function of *α*. We first consider a pessimistic scenario in which the expected returns on investment are low, yet higher than in the case of low volatility. The impact of administrative costs is expected to be similar to that in the low volatility investment case, because even if volatility is now higher, the mean return is also higher. We observe that the impact of administrative costs (*β*) in the medium volatility case is now lower than it was in the low volatility case. The impact in terms of the return lost due to administrative costs is roughly the same in the breakeven case as in that of the low volatility investment. But the optimistic scenario gives a much worse result than that of the low volatility situation.

It should be noted in Figure 5(b) that the proportion of wealth invested in stocks cannot be very large in this scenario if we want to be able to find the risk-adjusted equivalent in the presence of administrative costs. This means that if *α* becomes larger than the values shown, we find ourselves outside the parameter space and cannot calculate our risk-adjusted measure of lost return.

As we proceed to the high volatility scenario, Figures 6 and 7, we observe that *α* has a larger range but that the impact of administrative costs is also much higher here than in the pessimistic case, especially if *α* is low. In the most optimistic medium volatility scenario considered here, Figure 7, it can be seen that the time horizon should not exceed much more than eighty years; otherwise we can expect an enormous increase in *β* due to time persistence.

### 6.3. High Volatility

**Figure 8:**
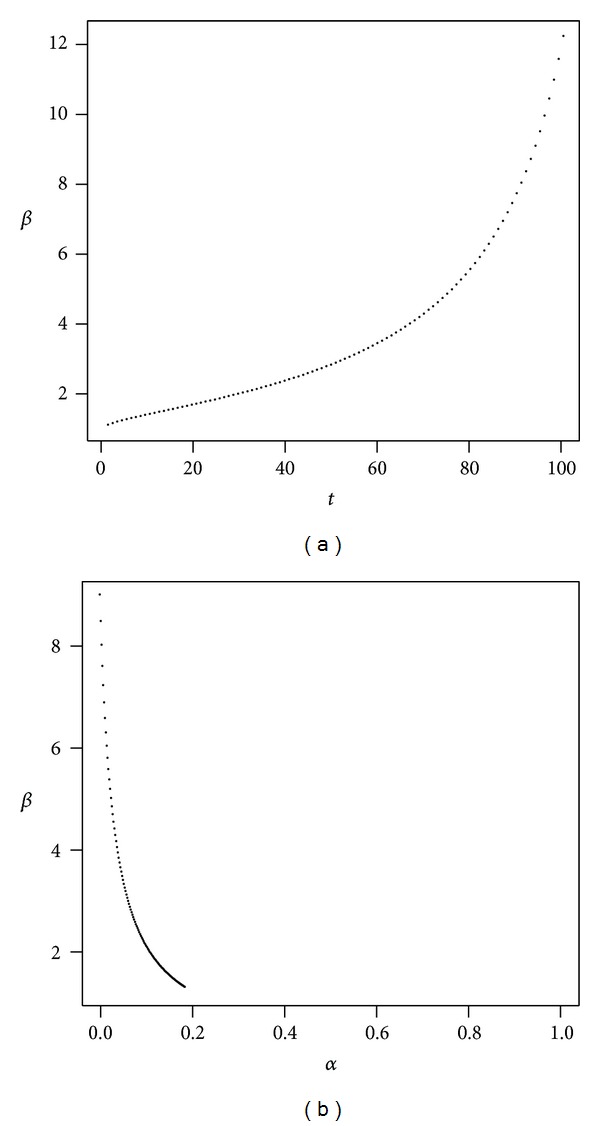
High volatility of returns (pessimistic). The plot shows *β* as a function of *t* with *α* = 50% (a) and *β* as a function of *α* with *t* = 40 (b). Parameters are *r* = 4%, *μ* = 17.5%, *σ* = 75%, *ν* = 3.1%, and *γ* = 10%.

**Figure 9:**
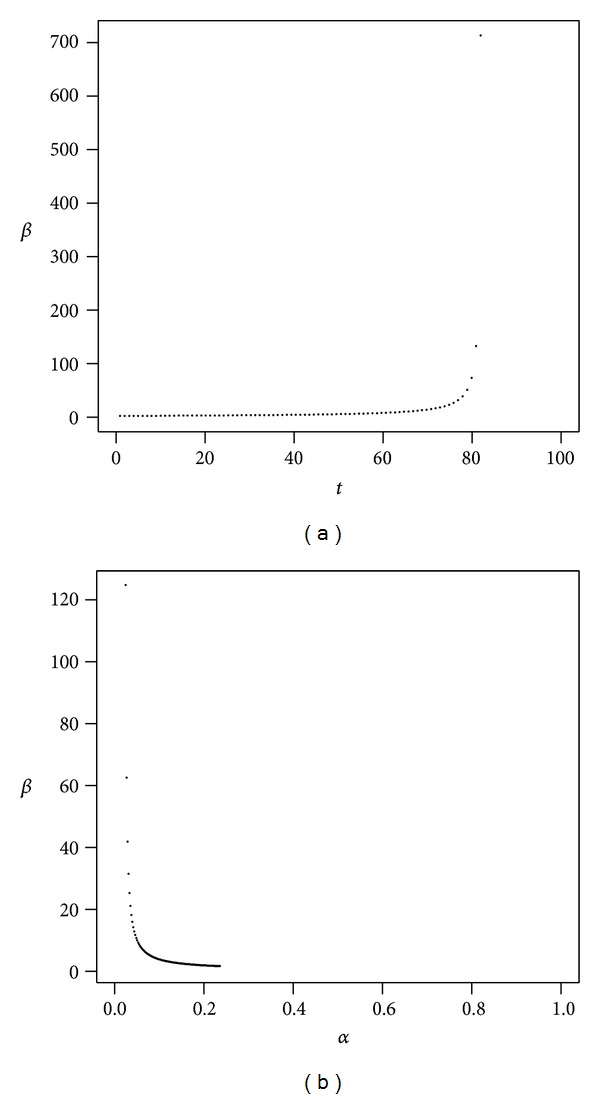
High volatility of returns (breakeven). The plot shows *β* as a function of *t* with *α* = 50% (a) and *β* as a function of *α* with *t* = 40 (b). Parameters are *r* = 4%, *μ* = 20.5%, *σ* = 75%, *ν* = 3.1%, and *γ* = 10%.

**Figure 10:**
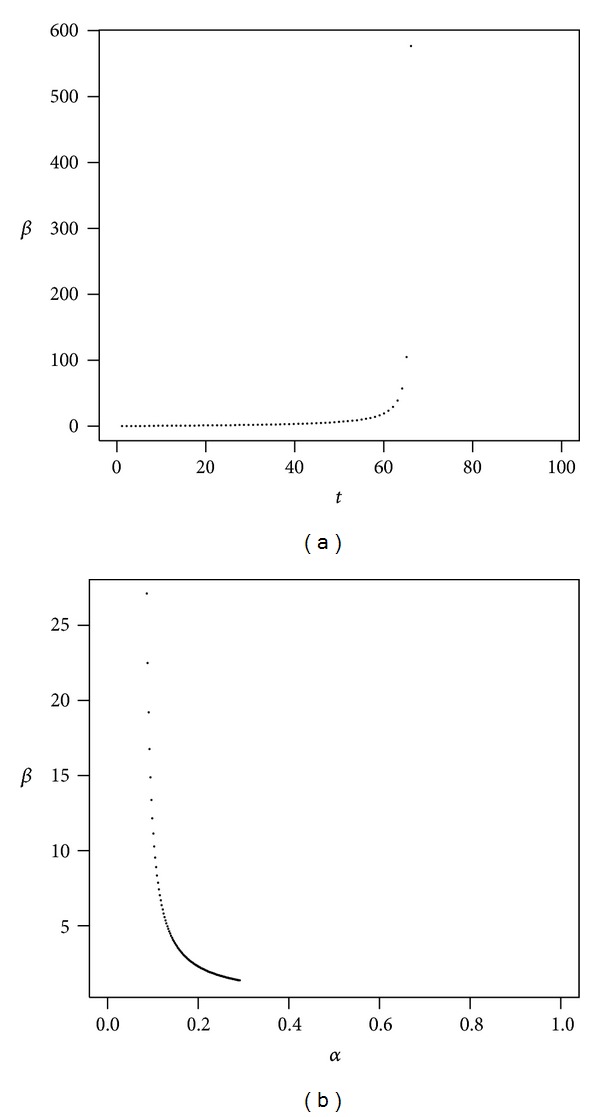
High volatility of returns (optimistic). The plot shows *β* as a function of *t* with *α* = 50% (a) and *β* as a function of *α* with *t* = 40 (b). Parameters are *r* = 4%, *μ* = 23.5%, *σ* = 75%, *ν* = 3.1%, and *γ* = 10%.

Long-term investments with high volatility typically also have high expected returns and, in general, incur high administrative fees. Figures 8, 9, and 10 present the evolution of *β* as a function of time and *β* as a function of *α* in the high volatility scenarios. In Table 1, where we have initially fixed *t* = 40 and a quantile probability equal to 10%, we see that for a high volatility investment we obtain the highest value of *β*, namely, 4.487 for the optimistic case. In Figures 9(a) and 10(a) it can be seen that the value of *β* tends to infinity as *t* approaches 80 and 66, respectively, which means that the impact of administrative costs on the reduction in the rate of return is extreme at that boundary.

In high volatility scenarios, the parameter space is fairly restrictive for the values of *α*, that is, the proportion invested in stocks, in order to find the risk-adjusted lost return due to administrative costs. Again our results indicate that the impact of administrative costs, even if the excess returns (*μ* − *r*) are large, is substantial when the volatility is high.

## 7. Concluding Remarks

Here we have highlighted the substantial impact administrative costs can have on investment plans with long-term horizons. On the understanding that an analysis limited to expected returns cannot provide a risk-adjusted evaluation of the rate of return that is lost as a result of administrative costs, we propose a one-step method to approximate the size of their impact. We illustrate our method with examples of realistic, standard fund types and, in many plausible scenarios, we often find the multiplier to be well above one, indicating that the impact of administrative fees is much greater than their actual size.

Our findings regarding the difference in the rate of return when introducing moderate administrative costs are much greater than expected; and it is our belief that this accounts for the poor performance of many common fund types. If administrative costs are fixed and are not dependent on performance, then they can be so highly detrimental to the long-term return that the investor is trapped in a clearly suboptimal investment strategy.

The simple approximation of the risk-adjusted impact can be used to show investors the importance of administrative costs and is a relevant contribution of this study in its own right. The only requirements to make this estimation are the time horizon, the volatility estimates, the excess returns, and the proportion of wealth invested in stocks.

Finally, in highly realistic scenarios, we have shown that the impact of administrative costs in terms of the approximate lost rate of return is often twice the amount of these administrative fees. This is particularly disturbing for those investing in pensions. We believe that our contribution can provide greater insights into the analysis of administrative costs and change perspectives regarding the information supplied by intermediaries when communicating to investors the possible impact of administrative fees. Underestimating the effects on lost returns is potentially very high, especially if investors erroneously believe the lost rate of return to be exactly equal to the administrative costs.

## Figures and Tables

**Figure 11:**
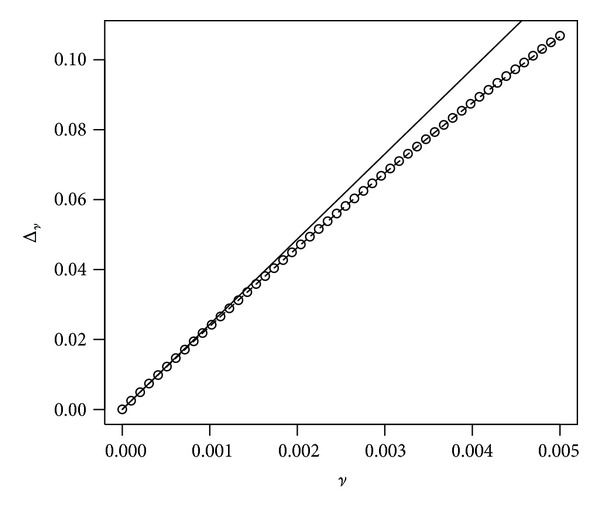
The plot shows Δ_*ν*_ as a function of *ν* (dots) with the first-order (solid line) and the second-order (dashed line) Taylor expansions superimposed. Parameters are *r* = 4%, *μ* = 8%, *σ* = 20%, *γ* = 10%, *α* = 50%, and *t* = 40.

**Table 1:** Opportunity cost of administrative costs (*β*) for selected combinations of parameter values (*γ* = 10%, *t* = 40).

Fund	*r*	*μ*	*σ*	*ν*	*α*	*β*
Example 1	4%	8%	0.2	0.1%	50% ∈ (0%, 97.5%)*	1.974
Low volatility	4%	7%	0.16	0.1%	50% ∈ (0%, 113.3%)*	2.130
Medium volatility						
Pessimistic	4%	8.5%	0.25	1.1%	50% ∈ (0%, 54.4%)*	1.373
Breakeven	4%	9.5%	0.25	1.1%	50% ∈ (6.9%, 70.4%)*	1.883
Optimistic	4%	10.5%	0.25	1.1%	50% ∈ (22.9%, 86.4%)*	2.996
High volatility						
Pessimistic	4%	17.5%	0.75	3.1%	8.31% ∈ (0%, 18.5%)*	2.385
Breakeven	4%	20.5%	0.75	3.1%	10.49% ∈ (2.3%, 23.8%)*	3.305
Optimistic	4%	23.5%	0.75	3.1%	13.67% ∈ (7.6%, 29.2%)*	4.487

∗The interval indicates the domain for parameter *α* according to the conditions in Section 3.2.

## Appendices

### A. Conditions on the Parameters

Our parameter space is defined by two conditions, to which we add one additional restriction that is required in the next section.

Condition 1 . We assume that

(A.1)
$$\begin{array}{c} 0\leq \nu \leq \frac{-\sigma d_{\gamma }}{\sqrt{t}}. \end{array}$$

On one hand, we assume that administrative costs cannot be negative; on the other, we require a maximum bound for administrative costs in order to be able to control the risk-return relationship. Parameter *d*_*γ*_, which denotes the *γ*-percentile of a standard normal distribution, tends to −*∞* when *γ* tends to 0. The right-hand inequality of Condition 1 will always be satisfied if we consider a quantile probability level *γ* that is sufficiently small, so in practice we can admit all values for nonnegative administrative costs, *ν*, provided we work with a suitable tolerance or quantile (probability) level. It follows from (A.1) that $\max\left(0,\left(\left((\mu -r)+\sigma d_{\gamma }\right)/\sqrt{t}\right)/\sigma^{2}\right)\leq \left(\left(\mu -r-\nu \right)/\sigma^{2}\right)$ and then we can establish the second condition in our parameter space.

Condition 2 . Consider

(A.2)
$$\begin{array}{c} \max\left(0,\frac{\left(\mu -r\right)+\sigma d_{\gamma }/\sqrt{t}}{\sigma^{2}}\right)\leq \alpha \leq \frac{\left(\mu -r-\nu \right)}{\sigma^{2}}. \end{array}$$

The boundaries in (A.2) are consistent as a result of (A.1). The lower bound for Condition 2 indicates that the median of the wealth process increases as the proportion invested in stocks increases. Equivalently, we want (5) to be an increasing function of *α*. The upper bound in Condition 2 follows from the fact that the quantile decreases when *α* increases, so that more wealth can be lost if a larger proportion is invested in stocks. Thus, we impose (4) as a decreasing function of *α*.
Condition 2 has a consequence for the time horizon for investors. (This bound is also useful when examining our first-order approximation to quantify the impact of administrative costs.) It readily follows from (A.2) that the investment time horizon cannot be infinitely long; that is,

(A.3)
$$\begin{array}{c} t\leq {\left[\frac{\sigma d_{\gamma }}{\left(\mu -r-\alpha \sigma^{2}\right)}\right]}^{2}. \end{array}$$

In order to be able to compare the quantiles of terminal wealth for the case involving no administrative costs (*ν* = 0) with those for the case with administrative costs and lower *α*, we need an additional condition to hold.

Condition 3 . Consider

(A.4)
$$\begin{array}{c} \frac{2\alpha \nu }{\sigma^{2}}\leq {\left[\frac{(\mu -r-\nu )+\sigma d_{\gamma }/\sqrt{t}}{\sigma^{2}}-\alpha \right]}^{2}. \end{array}$$

### B. First-Order Approximation Accuracy

In our example, we use the capital market parameters *r* = 4%, *μ* = 8%, and *σ* = 20%, which are similar to those used by other authors, including, for example, [32].

To assess the quality of the Taylor expansion, Figure 11 shows Δ_*ν*_ as a function of *ν* with the first- and second-order Taylor expansions superimposed. It can be seen that the second-order Taylor expansion provides very exact approximations for the values of *ν* considered here, while the first-order expansion is very exact for *ν* < 0.20% in our set of parameters.
